# A Single-Center 7-Year Experience with End-Stage Renal Disease Care in Nigeria—A Surrogate for the Poor State of ESRD Care in Nigeria and Other Sub-Saharan African Countries: Advocacy for a Global Fund for ESRD Care Program in Sub-Saharan African Countries

**DOI:** 10.1155/2012/639653

**Published:** 2012-06-28

**Authors:** Datonye Dennis Alasia, Pedro Emem-Chioma, Friday Samuel Wokoma

**Affiliations:** Renal Unit, Department of Internal Medicine, University of Port Harcourt Teaching Hospital, PMB 6173, Rivers State, Port Harcourt 50001, Nigeria

## Abstract

*Background*. A single-center ESRD care experience in a Nigerian teaching hospital is presented as a surrogate case to demonstrate the prevailing ESRD care situation in Nigeria and most SSA countries. *Methods*. The data of 320 consecutive ESRD patients undergoing maintenance haemodialysis treatment during a seven-year period were retrospectively analyzed. *Results*. Over 80% of the subjects funded dialysis treatments from direct out of pocket payment. The mean duration on dialysis before dropout was 5.2 ± 7.6 weeks, with majority 314 (98.1%) of the patients unable to sustain dialysis above 12 weeks. Total dialysis sessions during the 7-year period was 1476 giving an average weekly dialysis session of 0.013 (0.05 hour/week) per patient per week. One hundred and twenty-eight (40%) patients died within 90 days of entry into dialysis care. *Conclusions*. ESRD care in this single centre was characterized by gross dialysis inadequacy and case fatality due to the inability to access and afford care. The opportunities for kidney transplantation are also very low. Poverty and the absence of government support for ESRD care are responsible for the poor outcomes. A global focus on ESRD care in SSA countries has thus become imperative.

## 1. Introduction

The burden of end stage renal disease (ESRD) is increasing in Sub-Saharan African (SSA) countries [1, 2]. This is attributed to the rising prevalence of noncommunicable diseases [3] and infection-related nephropathies [2, 4].

In spite of this increasing burden, care for ESRD in SSA is suboptimal, compared with ESRD care in Europe and North America, where ESRD care is often in accordance with kidney disease quality-of-life guidelines (KDOQI) [5–7]. ESRD care in most of the SSA countries is characterized by gross lack of access to optimal care [5–8], with a rudimentary and unorganized institutional and human resource base for care. Virtually, all ESRD care inputs are imported into SSA countries and are thus quite expensive [5, 8]. The cost of care is also prohibitive and outside the reach of majority of the victims [2, 5, 8]. The bulk of the patients with ESRD belong to the lower socioeconomic group and are thus unable sustain maintenance dialysis for longer than three months of entry into the ESRD program [9]. Affordability and access to erythropoietin stimulating agents (ESA) is extremely poor while opportunities for kidney transplant are also limited [9, 10].

For the majority of SSA countries, there is hardly any, government-driven or government-supported ESRD care program. As a result, the mortality rate within 90 days of commencing renal replacement therapy (RRT) of ESRD patients in SSA countries is as high as over 90%, compared with patients in Europe and North America where it is less than 4% [9–11]

The objective of this paperis to use a single-center ESRD care experience in Nigeria, to demonstrate the prevailing lack of access to ESRD care in order to advocate the need for a global funding mechanism for ESRD populations in Sub-Saharan Africa.

## 2. Study Design and Methods

The demographic and clinical data of all consecutive patients, aged 15 years and above, who commenced maintenance haemodialysis treatment at the University of Port Harcourt teaching hospital, Port Harcourt, Nigeria between the January 1, 1996 to December 31, 1999 (4 years) and January 1, 2007 to December 31, 2009 (3 years), due to the interruption of dialysis services between January 1997 and December 2006 for technical problems, were retrospectively analyzed.

The diagnosis of ESRD was based on the KDOQI guidelines [12] for diagnosis and classification of CKD. The data for analysis included the biodata, overall clinical status, blood pressure measurements and the haematologic and biochemical indices (relevant to ESRD) at first presentation. Others included the duration of stay on the maintenance haemodialysis programme, the total number of haemodialysis sessions received during the period, and the average urea reduction ratio (URR) attained. Other parameters were the average Kt/V ratio attained before dropout, patient's actual annual incomes, and their sources of funding for ESRD care.

Four end points of the study were the proportion of the ESRD patients that were alive and still on the program at 3 months, 6 months, and 1 year from the date of entry into the ESRD program, the proportion that had dropped out of the program and lost to followup at the stated periods above, the proportion of who died at the given periods and referral for kidney transplant for the period under study.

### 2.1. Data Management

Statistical data were analyzed using Epi-info version 6.4. The literature search was done using manual search of relevant Nigerian and African medical journals and electronic search of medical research database through PubMed, HINARI, and google. The search keywords used were ESRD; RRT; outcomes and sub-Saharan Africa. Data was analyzed using descriptive statistics and presented as mean ± standard deviation and percentages.

## 3. Results

The data for 320 consecutive ESRD patients who satisfied the inclusion criteria were suitable for analysis. They consisted of 200 males and 120 females (*M*/*F* = 1.6 : 1), with a mean age of 46.2 ± 17.6 years, and age range 15–79 years (Table 1). They had a bimodal peak age pattern, with an early small peak in the 20–29 year age group and a second larger peak in the 50–59 and 60–69 year age groups, respectively.

The results of the evaluation of the income status of a cohort 40 ESRD patients in 2009 (Table 2) show that majority of the subjects were predominantly from the lower and middle socioeconomic classes, with 40% of them in socioeconomic classes V and VI, 45% in social classes III and IV and only 15% from the upper social classes I and II, as defined by the following income range based on annual family incomes. Lower or social classes V and VI earning 800 to 2,460 United States dollars (USD) or 0.12–0.4 million Nigerian naira (NGN); middle class or social classes III and IV earning 4,677 to 7,333 USD or 0.7 to1.2 million NGN; upper or social classes I and II earning 14,000.0 to 26,667 USD or 2.1 m to 4 million NGN. The prevailing exchange rate then was 150 NGN to 1 USD. The computed annual cost of haemodialysis treatment, per patient, during the period under study was 15,600.00 USD (2,340,000.00 NGN). The result shows the wide disparity between the annual incomes of the patients and the annual cost of haemodialysis treatment per patient.

**Figure 1:**
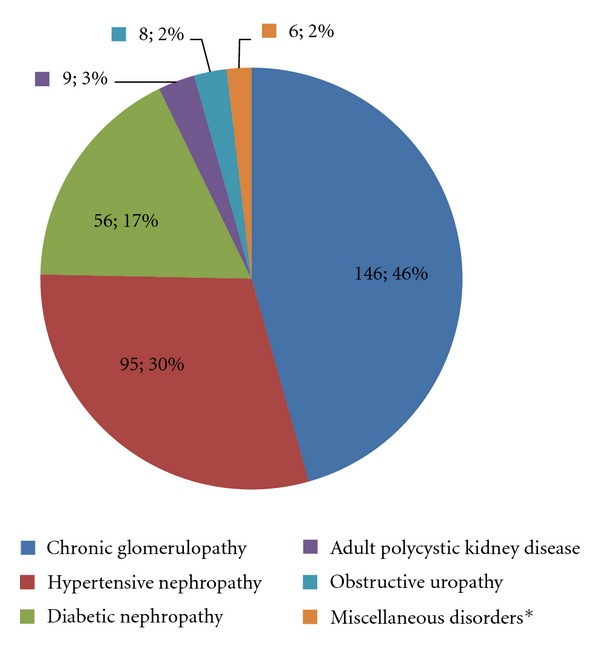
Chart showing causes of CKD in study subjects. *Miscellaneous disorders: Analgesic nephropathy(1), Multiple myeloma(1), chronic pyelonephritis(1), inadvertent nephrectomy(1), sickle nephropathy(1), and indeterminate(1).

Chronic glomerulopathy (45.6%), hypertensive nephropathy (29.7%), and diabetic nephropathy (17.5%) were the three leading causes of ESRD in the patients (see Figure 1). The diagnosis of the cause of CKD was clinical, as most of the patients presented in ESRD when a biopsy is of little value and determining if hypertension is a cause or effect of the kidney disease is difficult. However, the duration of hypertension, duration of diabetes, evidence of nephritis, the patient's age, and a history of prior renal disease such as acute glomerulonephritis (AGN) and ultrasound determined renal size were guides to determining the aetiology.

The patient's baseline hematologic and biochemical parameters are as outlined in Table 3.

Their mean e-GFR was 6.2 ± 5.8 mls/min/1.73 m^2^. Anaemia was found at presentation in over 90% of the subjects with a mean hematocrit (Hct) of 20.8 ± 6.8% and a range of 10–35%. Severe anaemia with Hct <21% was seen in 45.7% of subjects.

Their mean systolic and diastolic blood pressures were 171.2 ± 31.9 mmHg, range (107–240 mmHg) and 102.5 ± 27.4 mmHg, range 70–140 mmHg, respectively.

Using the JNC-7 criteria [13], 88.5% of the patients were hypertensive with 70.3% presenting with grade II hypertension.

At presentation, 85% of the patients were in an unstable clinical state, characterized by gross edema, anemia, severe hypertension, pulmonary edema, cardiovascular instability, and uraemic encephalopathy.

The duration of maintenance haemodialysis before loss to the program ranged from 1 to 37 weeks, with a mean duration of 5.2 ± 7.6 weeks. The longest dialyzing patient did so for a total of 37 weeks. This was a Nigerian ESRD patient resident in the United Kingdom visiting Nigeria.

Table 4 shows the distribution of the patients in accordance with the length of time spent in the dialysis program. Majority of the patients 314 (98.1%) could not sustain dialysis for more than 12 weeks. Only three patients (0.9%) could sustain dialysis for over 26 weeks before they were lost to followup.

All patients achieved an aggregate mean urea reduction ratio (URR) of 48.7 ± 22%, range 8–88% and an aggregate mean Kt/V of 0.94 ± 0.4, range 0.5–1.9, respectively.

The 320 patients received a total of 1,476 haemodialysis sessions during the seven-year period under consideration, translating to an average weekly dialysis rate of 0.013 dialysis sessions (0.05 hour per week) per patient compared to the expected 3 sessions (12 hours) per week per patient.

Most of the patients 285 (89.1%) could only achieve an average of 1–9 dialysis sessions before dropping out of the program, while the remaining 35 (9.9%) achieved an average of 10 to 20 dialysis sessions before dropping out of the program.

Within 12 weeks of commencing maintenance dialysis, 97.8% of the patients had dropped out of the program through deaths and abandonment. Forty percent of this number were confirmed dead, 41.8 percent absconded and were presumed dead, making a total of 81.8% deaths within 12 weeks of entry into the program. Eight patients (2.5%) who were able to raise funds were at various times referred out of the country for renal transplant. Only patients who had kidney transplants survived beyond six months.

**Figure 2:**
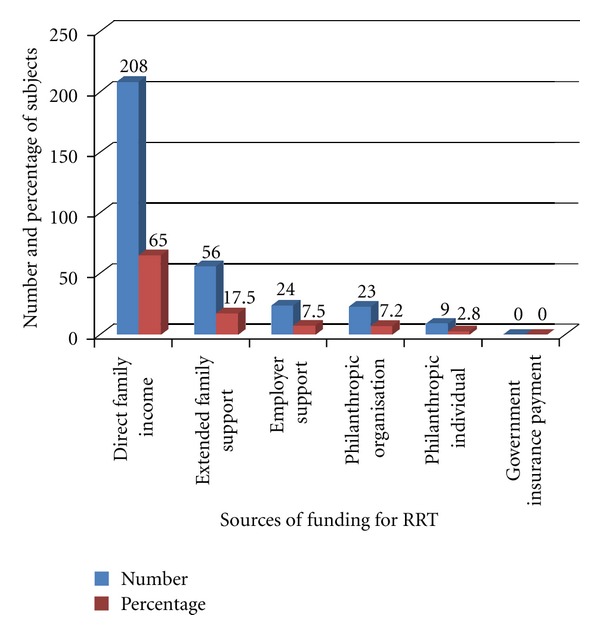
Chart showing sources of funding for ESRD care.

Analysis of the source of funding for ESRD care in the subjects showed that 65% of these patients funded their maintenance dialysis treatment from direct out of pocket payment, 17.5% received some support from extended family sources to fund treatment, while the remaining 17.5% of the patients obtained some support from philanthropic individuals or organizations such as church groups. None of the patients were covered by insurance payments, and there was no government subsidy for the payment of maintenance dialysis of the patients (Figure 2).

There was no difference in outcomes when the two periods under review were compared.

## 4. Discussion

The results from this single-center ESRD care experience show that ESRD patients in our center are predominantly young adults and middle-aged people from the lower socioeconomic group with abysmally poor access to ESRD care, grossly suboptimal ESRD care, and minimal access to kidney transplant.

Their incomes fall far below the cost of ESRD care, resulting in a 90-day mortality rate of almost 100% from the point of entry into the ESRD program. The dominant factors for this poor outcome are economic deprivation and the lack of structured government socioeconomic support.

This pattern is consistent with single-center experiences in other centers in Nigeria and other SSA and African countries with the exception of South Africa. Menakaya et al. [14] in a retrospective study of 454 ESRD patients on maintenance dialysis, during a ten-year period (1981–1994) at the Lagos university teaching Hospital (LUTH), southwestern Nigeria, recorded 29.7% in hospital mortality with 65% percent of cases abandoning the treatment and subsequently presumed dead within three months of entry into the ESRD the program. Arogundade et al. [15], in Ile-Ife, Southwestern, Nigeria, reported 77.8% mortality within 3 months of commencing maintenance dialysis in 540 ESRD patients over a period of 15 years. Similarly, Chijioke et al. [16] and Bosan and Ibrahim [17] in two different teaching hospitals in the northern parts of Nigeria reported mortality rates of 66.7% and 90.8% respectively, all within three months of entry into maintenance dialysis program; while Arodiwe et al. [18] in Enugu, southeastern Nigeria reported a mortality rate of 67.3% within three months of entry. In all these studies, none of the ESRD patients was able to sustain maintenance dialysis for longer than six months.

Available data from other SSA countries indicate the gross paucity of kidney care facilities and very poor access to ESRD care. The number of patients on dialysis in some SSA countries, shown in a 2003 report [8], indicates the following number of patients per country: Mauritania (50), Senegal (20–30), Mali (20), Burkina Faso (18), Ivory Coast (130), and Ghana (30), respectively. Others are Zimbabwe (59), Kenya (100), and Sudan (200). South Africa is the only SSA country with a more reliable ESRD data and much higher access to organized ESRD care. By 1998 [5], South Africa had 1525 ESRD patients registered in the South African Renal dialysis and transplantation registry (SADTR) on regular dialysis with ESRD-care financed by the government.

The data shown above makes it evident that hundreds of thousands of ESRD patients in most SSA countries have practically no access to effective ESRD care, compared with their counterparts in the developed countries. The over 90-percent case fatality rate within 90 days of diagnosis of ESRD is unacceptable in the present state of knowledge and global development in nephrology care. Furthermore, the direct and indirect human and socioeconomic cost of the high mortality associated with ESRD in SSA will continue to affect the economy of SSA countries adversely.

The prevailing situation of ESRD care in SSA described above is a challenge for the global renal community, as action is required to prevent an expected worsening, in view of the increasing incidence and prevalence of CKD and ESRD. This situation thus calls for strategies that could be adopted to improve the situation.

Short periods of maintenance dialysis and early transplant for ESRD patients in SSA countries has been advocated [5, 8] in response to the current situation. As reasonable as this option may be, the prevailing experience does not support this option. Firstly, even in the developed countries, less than 50% of ESRD patients are transplanted as data from the United States and European renal transplant registries indicate that only 16% to 48% of ESRD patients on the transplant waiting list get transplanted per year [19, 20]. The reasons for the relatively low transplant rate include the shortage of donor kidneys for transplant, HLA- compatibility problems as well as some medical morbidity that may contraindicate transplant [21, 22].

Secondly, the same reason of high cost, which prevents access to maintenance dialysis, will also prevent access to transplant in SSA countries. From experience in Nigeria, live-donor kidney transplant costs between 7 and 10 million NGN (33,000.0–76,000.0 USD) excluding costs of immunosuppressive agents. Therefore, in the absence of any government support only very few persons can access live-donor transplant in Nigeria. This situation can be extrapolated for most SSA-countries, considering the existing high poverty rate in these countries. Thirdly, getting both related and unrelated live donors in SSA countries is difficult due to cultural barriers, religious beliefs, and lack of monetary incentives for donors among other reasons. Finally in most SSA countries' health systems do not have capabilities for nonheart beating kidney donations and are not likely to do so in the foreseeable future.

Resultantly, it is evident that early live-donor kidney transplant program may not provide an adequate solution to the grim realities facing ESRD patients in SSA countries.

As is the practice in the developed countries of the world, maintenance dialysis through CAPD and or Haemodialysis remains the cornerstone of ESRD care for the following reasons. Not all eligible ESRD patients can benefit from transplant; therefore, the rest must remain on maintenance dialysis to keep alive. For reasons such as age, debility, certain comorbid disorders, and HLA-incompatibility [21, 22], not all ESRD patients are suitable candidates for renal transplant. More so patients with graft failure will return to maintenance dialysis until another suitable kidney can be found. Therefore, all countries irrespective of economic status must necessarily have viable and sustainable maintenance dialysis programs.

It is certain that individual patients cannot afford self-funding of ESRD care in SSA countries as was the case for developed countries before now. It was in realization of this fact that the American Congress in 1973 passed the legislation that led to the birth of an End-Stage Renal Disease (ESRD) Program, which was later followed by the US organ transplant Act of the US Congress that enabled ESRD patients benefit from kidney transplant [23, 24]. The annual estimate of government expenditure for ESRD care in developed countries also justifies this as the “US government spent about $12.04 billion in annual Medicare spending on ESRD in 1998, not including an additional out-of Medicare $4.7 billion spending by employer group health plan for the sustenance of the ESRD program” [25].

It stands to reason that if ESRD patients in SSA-countries are to benefit from effective ESRD care there should be some mechanisms in place to enable all ESRD patients have access to optimal ESRD care in order to improve the survival and quality of life. Two strategies are thus proposed, and their potential strength and weaknesses are appraised.

### 4.1. The SSA Country Self-Reliance Strategy

The SSA country self-reliance strategy would involve the SSA countries taking the initiative and challenge to institute their own models of ESRD care that will enable universal access to optimal ESRD care of their citizenry. Some emerging countries like Brazil and Singapore [26, 27] have established mechanisms that ensure universal access to ESRD care for their populations. While the Brazilian government provides ESRD care free to all affected persons, Singapore adopted a public-private partnership option in realizing the same objectives. In both countries, there is the political will by the governments to ensure universal access to optimal ESRD care for their citizenry.

Few countries in the SSA which are relatively richer such as Nigeria may be able to adopt the same strategy with adequate political will by government. It would be expected that a country like Nigeria that earned an average of 59 billion USD in 2010 from crude oil export sales [28] should have the capacity to make ESRD care universally accessible for its citizenry; however, this is not the case. The maintenance dialysis data in Nigeria as shown above is a clear indication of the lack of political will by the government. The Nigerian scenario is also is prevalent in several other SSA countries.

In addition to the lack of political will by governments in most SSA countries, other political, social, and economic factors which impede optimal ESRD care such as the high poverty rate among the populace, the reliance on importation of almost all ESRD care products, the relative lack of human capacity, and lack of reliable health data for planning also pose enormous challenges.

In the present political and economic state of affairs in most SSA countries highlighted above, it is very doubtful if the hindrances can be addressed in the projected future on the short and medium terms. For this reason, the self-reliance option may not feasible.

### 4.2. ISN-Driven Global Fund Initiative for ESRD Care in SSA Countries

The second strategy proposed is the institution of an international society of nephrology (ISN) and or WHO-driven global fund for comprehensive ESRD care programs in SSA countries. Such a fund could be patterned along the lines of the global funds initiative for HIV/AIDS, tuberculosis and malaria [29] with modifications as necessary. The fund should be a counterpart fund in which the international global contributors, host SSA country governments, ESRD-care products industry, as well as the ESRD patients and families shall be stake holder contributors.

The contributions of the ESRD care industry can be in the form of donations of some percentage of their annual operational profits or turnover or through price discounts on their products. Host SSA countries could also create incentives for these industries to establish production factories for some of their products in host SSA countries. Other corporate concerns such as petroleum and major manufacturing companies doing business in host SSA countries can be encouraged to contribute to the fund as part of their community and corporate social responsibilities.

 ESRD patients shall contribute through payments of subsidized fee for service either directly or through insurance arrangements. However, it will be important to develop mechanisms which ensure that truly indigent and disadvantaged ESRD patients could be treated for free to ensure universal access and lack of discrimination.

The ISN and the SSA country regional kidney associations and foundations should serve as facilitators and regulators of the program to ensure strict compliance by host SSA countries to the terms of engagement.

The proposed fund should be disbursed in appropriate proportions to cover direct patient treatment (dialysis, transplants, and immunosuppressive agents), human capacity development for renal care, equipment and infrastructural developments as well as advocacy and the prevention of CKD and research.

The global fund for the treatment and control of HIV/AIDS, tuberculosis, and malaria has achieved tremendous success since its inception in 2005. The prevalence of HIV/AIDS in most global funds beneficiary SSA countries are on the decline. In Nigeria, the HIV prevalence has dropped from 5.8% in 2001 to 4.0% in 2006 [30]. Data from the United Nations against AIDS (UNAIDS) showed that by 2006 an estimated 1.3 million people in SSA countries were on antiretroviral therapy (ART). At the end of 2 years of commencing ART, 60% of the HIV/AIDS patients on ART were alive. The UNAIDS states that “three years after the creation of the global fund it is proving to be the engine behind the scale up of the fight against the pandemic worldwide. Fatalism, inevitability and stigma are being replaced with hope and greater openness about HIV/AIDS” [31].

Such a global fund mechanism could also promote wider access to ESRD care, develop needed the capacity for kidney disease care, enhance renal care infrastructure as well as promote prevention of CKD in the SSA countries.

This option seems to provide hope for sustainability as the SSA countries may be left to take on the responsibility for the program on the long term using the already established framework of the global initiative.

## 5. Conclusion

ESRD patients in SSA countries have very minimal access to optimal care as a result of poverty, very low capacity for ESRD care, and the absence of any government input in the care of ESRD. This has resulted in an above one-year mortality rate after diagnosis of almost 100%.

SSA countries in the present state of poor governance, weak economies, poor economic management, and seeming lack of proper direction and priority setting, have not demonstrated any willingness to intervene in ESRD care as their counterparts in the developed countries and some emerging countries.

An ISN-driven global fund initiative for ESRD care in SSA countries, as is the case with the global fund for HIV/AIDS which has shown significant success is recommended. Global partners, SSA host countries, ESRD care products industry, ESRD patients, and ISN/regional kidney societies shall constitute the stake holders.

The global fund strategy will aim to provide direct ESRD care, human and infrastructural capacity development for CKD care as well as CKD preventive programs.

It is expected that this strategy will change the prevailing unacceptable outcomes of ESRD care in SSA.

## Figures and Tables

**Table 1:** Showing the age distribution of subjects.

Age group (years)	Number of subjects	Percentages
10–19	40	12.6
20–29	55	17.2
30–39	44	13.8
40–49	48	14.9
50–59	60	18.9
60–69	59	18.4
70–79	7	2.3
>80	7	2.3

Total	320	100

**Table 2:** Annual income data for a cohort of 40 subjects.

Income group	Social class	Number	Percentage
I-II (14000–26667 USD per annum)	Upper	6	15
III-IV (4677–7337 USD per annum)	Middle	16	40
V-VI (800–2460 USD per annum)	Lower	18	45

		40	100

**Table 3:** Baseline characteristics of study subjects at first presentation.

Baseline variable	Mean (Standard deviation)	Range
e-GFR (mL/min/1.73 m^2^)	8.2 (5.8)	2.7–12.5
Haematocrit (%)	20.8 (6.8)	10–35
Sodium (mmol/L)	134.8 (6.0)	122–149
Potassium (mmol/L)	4.7 (1.0)	3–7
Bicarbonate (mmol/L)	19.4 (7.8)	8–27
Urea (mmol/L)	32.9 (36.2)	14–56.3
Creatinine (*μ*mol/L)	1224.9 (557.4)	245–2505
Total protein (g/dL)	60.7 (4.0)	57–65
Albumin (g/dL)	32.0 (9.8)	25–39
Systolic blood pressure (mmHg)	171.2 (31.9)	107–240
Diastolic blood pressure (mmHg)	102.5 (27.4)	70–140

**Table 4:** Haemodialysis exposure status of subjects.

Duration of haemodialysis exposure (weeks)	Number of subjects	Percentage
1–12	314	98.1
13–24	3	0.9
25–36	3	0.6
37–52	1	0.3
>52	—	—

## References

[1] Barsoum RS (2006). Chronic kidney disease in the developing world. *New England Journal of Medicine*.

[2] Sumaili EK, Cohen EP, Zinga CV, Krzesinski JM, Pakasa NM, Nseka NM (2009). High prevalence of undiagnosed chronic kidney disease among at-risk population in Kinshasa, the Democratic Republic of Congo. *Nephrology Dialysis Transplantation*.

[3] National Expert Committee on communicable diseases (NCD) Non communicable diseases, final report of a National survey.

[4] Akinsola W, Odesanmi WO, Ogunniyi JO, Ladipo GOA (1989). Diseases causing chronic renal failure in Nigerians—a prospective study of 100 cases. *African Journal of Medicine and Medical Sciences*.

[5] Naicker S (2003). End-stage renal disease in sub-Saharan and South Africa. *Kidney International*.

[6] Ansell D (2008). Summary of findings in the 2008 United Kingdom renal registry report. *The 11th Annual Report*.

[7] United States Renal Data System (USRDS) Annual Data report Bethseda Md. National Institute of Diabetes, Digestive and Kidney.

[8] Bamgboye EL (2003). Hemodialysis: management problems in developing countries, with Nigeria as a surrogate. *Kidney International*.

[9] Odutola TA, Ositelu SB, D’Almeida EA, Mabadeje AFB (1989). Five years experience of haemodialysis at the Lagos University Teaching Hospital (November 1981 to November 1986). *African Journal of Medicine and Medical Sciences*.

[10] Arije A, Kadiri S, Akinkugbe OO (2000). The viability of hemodialysis as a treatment option for renal failure in a developing economy. *African Journal of Medicine and Medical Sciences*.

[11] Isikiru D, Jones EHP, Briggs JD (1999). Death within ninety days of starting renal replacement therapy in the ERA-EDTA registry before 1990 and 1992. *Nephrology Dialysis Transplantation*.

[12] National Kidney Foundation (NKF/DOQI) (2002). Clinical practice guidelines for chronic kidney disease: evaluation, classification and stratification. *American Journal of Kidney Diseases*.

[13] Chobanian AV, Bakris GL, Black HR (2003). Seventh report of the joint National Committee on Prevention, Detection, Evaluation, and Treatment of High Blood Pressure. *Hypertension*.

[14] Menakaya NC, Adewunmi AJ, Braimoh RO, Mabayoje F (2006). End stage renal disease at the university of Lagos teaching hospital. A ten year update review. *Tropical Journal of Nephrology*.

[15] Arogundade FA, Sanusi AA, Akinsola A (2006). Epidemiology and clinical characteristics and outcomes on ESRD patients in Nigeria: is there a changing trend?. *Tropical Journal of Nephrology*.

[16] Chijioke A, Aderibigbe A, Rafiu MO, Olarewaju TO, Makusidi AM (2010). The assessment of haemodialysis adequacy among ESRD patients in Illorin using urea reduction ratio (URR). *Book of Abstracts-Nigeria Asssociation of Nephrology Conference*.

[17] Bosan IB, Ibrahim A (2007). The challenges of Renal dialysis in Ahmadu Bello University teaching hospital, Zaria. *Tropical Journal of Nephrology*.

[18] Arodiwe EB, Ijoma CK, Ulasi II, Onodugo OO (2010). Case fatality among CKD patients in University of Nigeria teaching hospital. *Book of Abstracts-Nigeria Asssociation of Nephrology Conference*.

[19] http://optn.transplant.hrsa.gov/.

[20] (2006). ERA-EDTA Registry. *Annual report*.

[21] Ansell D Summary of findings in the 2008 United Kingdom Renal registry Report.

[22] Gjertson DW, Terasaki PI, Takemoto S, Mickey MR (1991). National allocation of cadaveric kidneys by HLA matching—projected effect on outcome and costs. *New England Journal of Medicine*.

[23] Rettig RA (1991). Origins of medicare disease entitlements, social security amendments of 1972. *Biomedical Politics*.

[24] Idem (1986). *Organ Transplantation: Issues and Recommendations*.

[25] Xue JL, Ma JZ, Louis TA, Collins AJ (2001). Forecast of the number of patients with end-stage renal disease in the United States to the year 2010. *Journal of the American Society of Nephrology*.

[26] Zatz R, Romao JE, Noronha IL (2003). Nephrology in Latin America, with special emphasis on Brazil. *Kidney International*.

[27] Ramirez SPB, Durai TT, Hsu SIH (2003). Paradigms of public-private partnerships in end-stage renal disease care: The National Kidney Foundation Singapore. *Kidney International*.

[28] http://www.cenbank.org/Rates/.

[29] http://www.theglobalfund.org/...reports/Publication_2011Results_Report_e.

[30] http://naca.gov.ng/content/view/423/lang,en/.

[31] http://www.unaids.org/en/dataanalysis/epidemiology/2009aidsepidemicupdate/.

